# Get control of your commas

**DOI:** 10.1007/s40037-015-0248-y

**Published:** 2016-01-26

**Authors:** Lorelei Lingard

**Affiliations:** Schulich School of Medicine & Dentistry, Western University, Health Sciences Addition, Rm. 112, N6A 5C1 London, ON Canada

In the writer’s craft section we offer simple tips to improve your writing in one of three areas: Energy, Clarity and Persuasiveness. Each entry focuses on a key writing feature or strategy, illustrates how it commonly goes wrong, teaches the grammatical underpinnings necessary to understand it and offers suggestions to wield it effectively. We encourage readers to share comments on or suggestions for this section on Twitter, using the hashtag: #how’syourwriting?

Please start cutting, Dr. Franklin.

Please start cutting Dr. Franklin.

Comma placement can radically alter the meaning of a sentence. But many of us struggle to know where exactly to put them. How do you decide? Do you treat commas like salt, sprinkling them over your writing according to your personal taste? Have you a vague sense that, like too much salt, too many commas are bad for you? Or are you an adherent of the ‘breathing’ rule, inserting commas wherever a reader might need an O_2_ break? Have you ever wondered why those editing your work have removed one comma but not another?

The purpose of a comma is to separate clauses within a sentence, phrases within a clause or words within a phrase, in order to succinctly and unambiguously express meaning. Seems straightforward, right? Wrong. The comma is arguably the most misunderstood of punctuation tools. Ask someone about comma rules and even those who begin with confidence are likely to trail off apologetically. This is because comma use is not fully explained by rules; it depends in part on taste. But, as David Crystal insists in his history of punctuation, variation in comma use is neither infinite nor totally idiosyncratic [1]. It turns out that there are two broad schools of punctuation, and understanding them can help us to unravel the complexities of comma use. In the elocutional school, with its origins in antiquity, commas indicate intonation and pauses in oral speech. In the grammatical school, which arose with the advent of the printing press, commas express grammatical relations among parts of the sentence. What’s tricky is that both approaches are still alive and well, so that most of us have been trained, explicitly or implicitly, to use a bit of both in our writing.

Getting control of your commas requires distinguishing between rules and preferences, which map closely onto the grammatical and elocutional schools of comma use. The aim of this Writer’s Craft section is to help you ascertain when commas are prohibited, when they are necessary, and when they are unnecessary but acceptable as a matter of preference.

## Comma rules

This list is not exhaustive, nor is it in any particular order of priority. Based on my experience reviewing and mentoring scholarly writing in medical education, these are among the most common comma errors in our community.

1. When the *subject* and its **verb** are side by side, never separate them with a comma.

Incorrect: *Fourth-year medical students and first-year residents*, **participated** *in the study*.

Correct: *Fourth-year medical students and first-year residents* **participated** *in the study*.

If you are inclined to place a comma between your subject and verb, this usually signals that your subject is too long and the sentence should be reworked to shorten it. Inserting an elocutionary comma in this case—a comma that creates a pause so the reader can absorb the long subject and note the coming verb—is incorrect.

2. When a list follows a **verb**, there should not be a comma separating the verb from the list:

Incorrect: *The factors influencing residents’ decisions about formal research training* **are**, *debt, role models, program culture and personal ambition*.

Correct: *The factors influencing residents’ decisions about formal research training* **are** *debt, role models, program culture and personal ambition*.

3. Commas are not used on their own to join independent sentences.

Incorrect: *I came to medical school, I saw all the work I had to do, incredibly I conquered it*.

Correct: *I came to medical school. I saw all the work I had to do. Incredibly, I conquered it*.

4. In a compound sentence, a comma is necessary before the **conjunction** that joins the two independent clauses.

Incorrect: *The faculty agreed to participate in the new assessment scheme* **but** *they were not enthusiastic about its chances of success*.

Correct: *The faculty agreed to participate in the new assessment scheme*, **but** *they were not enthusiastic about its chances of success*.

5. In compound sentences that use a **conjunctive adverb**, a semi-colon should precede the adverb and a comma follow it:

Incorrect: *The faculty agreed to participate in the new assessment*, **however** *they were not enthusiastic about its chances of success*.

Incorrect: *The faculty agreed to participate in the new assessment*, **however**, *they were not enthusiastic about its chances of success*.

Correct: *The faculty agreed to participate in the new assessment*; **however**, *they were not enthusiastic about its chances of success*.

6. A comma is necessary between the main clause and subordinate clause in a complex sentence, regardless of which clause comes first:

Incorrect: *Although the new electronic patient record is not believed to influence medical teaching there has been no systematic study of its educational effects*.

Correct: *Although the new electronic patient record is not believed to influence medical teaching, there has been no systematic study of its educational effects*.

Correct: *There has been no systematic study of the new electronic patient record’s educational effects, although it is not believed to influence medical teaching*.

7. With relative clauses, a comma signals that the detail is parenthetical, while no comma indicates that the detail is necessary to the meaning of the sentence.

Parenthetical: *The faculty, who had been carefully trained, were expert clinical teachers*.

Necessary: *The faculty who had been carefully trained were expert clinical teachers*.

In the parenthetical version, all faculty had been carefully trained and all were expert teachers. You can imagine replacing the commas with brackets in this version. In the necessary, or ‘restrictive relative’ version, only the faculty who had been carefully trained were expert clinical teachers; the implication is that other faculty who were not carefully trained were not expert clinical teachers,

## Comma preferences

Editorial preferences for comma use abound. These preferences have evolved across history, with a more modern, minimalist approach to commas prevailing now in comparison with a 100 years ago [1]. Complicating matters, comma preferences are often presented as if they were rules. These commas, however, are not obligatory. In deciding whether to use them, you will usually be weighing their effect on elocution: do they help the reading process? According to Crystal, two factors are important in such decisions: 1) the length of the phrase to be separated and 2) the tightness of its semantic link with the rest of the sentence. The longer the length, the more likely the comma. The tighter the semantic link, the less likely the comma. (As Crystal acknowledges, the trouble is when these two factors are in tension, but that is beyond the scope of this brief paper.) [1]

Commas can be used, or not, to set off ‘wind-ups’ [2], those introductory phrases at the head of the sentence:

*In this qualitative research study, the focus was residents’ experiences of workplace-based assessment*.

*In this qualitative research study the focus was residents’ experiences of workplace-based assessment*.

*In this qualitative research study conducted by Sinclair et al during the shift to competency-based medical education in Canada, the focus was residents’ experience of workplace-based assessment*.

The last, longer wind-up is much easier to read with an elocutionary comma separating it from the main clause, and some would argue that this comma is grammatically obligatory. Crystal offers, as a general rule, that phrases longer than 7 words are more likely to take a comma in such cases [1].

Another matter of preference is whether there should be a comma before the conjunction joining the last two items in a list—called the ‘serial’ or Oxford comma:

*The committees requiring new members include occupational safety, finance, promotion and tenure, and strategic planning*.

Newspaper articles rarely use this comma; academic writing often does, but it varies by publishing press. When in doubt, check the usage for the journal you are writing for.

If you find yourself inserting many elocutionary commas to create pauses and ease reading in a sentence, this is likely a signal that your sentence is too long:

*Before embracing subjectivity, increasingly recognized as a key dimension of rater cognition, the medical education community needs to consider how subjectivity and objectivity interact and, even more critically, how current assessment instruments are structured to prompt, or inhibit, particular rater responses*.

This sentence breaks none of the comma rules outlined above. In total, however, this pile of commas signals a sentence that is overly complex and meandering. Don’t be afraid to break up such cumbersome constructions into a series of shorter, clearer sentences. This version is better:

*Subjectivity is increasingly recognized as a key dimension of rater cognition. Before the medical education community embraces subjectivity, however, it should consider how subjectivity and objectivity interact. Even more critically, it should consider how current assessment instruments prompt or inhibit particular rater responses*.

In summary, comma use baffles many writers. If you know the basic comma rules and the logic that governs comma preferences, you can better decide when a comma is prohibited, necessary, or a matter of personal taste. Consistency in these decisions is critical to ensuring clarity in your writing.
